# Are Asians comfortable with discussing death in health valuation studies? A study in multi-ethnic Singapore

**DOI:** 10.1186/1477-7525-4-93

**Published:** 2006-12-05

**Authors:** Hwee-Lin Wee, Shu-Chuen Li, Feng Xie, Xu-Hao Zhang, Nan Luo, Yin-Bun Cheung, David Machin, Kok-Yong Fong, Julian Thumboo

**Affiliations:** 1 Department of Rheumatology and Immunology, Singapore General Hospital, Republic of Singapore; 2 Department of Pharmacy, Faculty of Science, National University of Singapore, Republic of Singapore; 3 Health Services Research Unit, Yong Loo Lin School of Medicine, National University of Singapore, Republic of Singapore; 4 MRC Tropical Epidemiology Group, London School of Hygiene and Tropical Medicine, London, UK; 5 Clinical Trials & Epidemiological Sciences, National Cancer Centre Singapore, Republic of Singapore; 6 Clinical Trials & Epidemiology Research Unit, Republic of Singapore; 7 School of Health and Related Research, University of Sheffield, Sheffield, UK; 8 Department of Medicine, Yong Loo Lin School of Medicine, National University of Singapore, Republic of Singapore

## Abstract

**Background:**

To characterize ease in discussing death (EID) and its influence on health valuation in a multi-ethnic Asian population and to determine the acceptability of various descriptors of death and "pits"/"all-worst" in health valuation.

**Methods:**

In-depth interviews (English or mother-tongue) among adult Chinese, Malay and Indian Singaporeans selected to represent both genders and a wide range of ages/educational levels. Subjects rated using 0–10 visual analogue scales (VAS): (1) EID, (2) acceptability of 8 descriptors for death, and (3) appropriateness of "pits" and "all-worst" as descriptors for the worst possible health state. Subjects also valued 3 health states using VAS followed by time trade-off (TTO). The influence of sociocultural variables on EID and these descriptors was studied using univariable analyses and multiple linear regression (MLR). The influence of EID on VAS/TTO utilities with adjustment for sociocultural variables was assessed using MLR.

**Results:**

Subjects (n = 63, 35% Chinese, 32% Malay, median age 44 years) were generally comfortable with discussing death (median EID: 8.0). Only education significantly influenced EID (p = 0.045). EID correlated weakly with VAS/TTO scores (range: VAS: -0.23 to 0.07; TTO: -0.14 to 0.11). All subjects felt "passed away", "departed" and "deceased" were most acceptable (median acceptability: 8.0) while "sudden death" and "immediate death" were least acceptable (median acceptability: 5.0). Subjects clearly preferred "all-worst" to "pits" (63% vs. 19%, p < 0.001).

**Conclusion:**

Singaporeans were generally comfortable with discussing death and had clear preferences for several descriptors of death and for "all-worst". EID is unlikely to influence health preference measurement in health valuation studies.

## Background

Health valuation studies are performed to understand population preferences for various health states and are important in informing healthcare resource allocation [1]. The topic of death is invariably raised in such studies as subjects are required to value death either directly, for example, when visual analogue scales (VAS) are used or indirectly, for example, when time trade-off (TTO) or standard gamble (SG) are used [2]. Previous studies have found that health preferences may be influenced by respondent characteristics such as age [3], social class [4], educational status [4] and presence of illness [5,6]. However, to the best of our knowledge, no studies have investigated how willingness to discuss death may affect health preference measurements in health valuation studies.

Reluctance to discuss death may potentially reduce participation in health valuation studies, thus resulting in selection bias. It may also increase the prevalence of missing valuation data for the health state of being dead, which is particularly problematic because these values are required to rescale raw scores onto a 0 (dead) to 1 (perfect health) scale [7]. These missing values would also render other associated data unusable, resulting in significant data wastage [8]. Reported prevalence of missing dead valuations ranged from 8% to 71% [8-10]. Reluctance to discuss death may be particularly relevant in an Asian population, where, for example, many Japanese and Chinese avoid talking about death because they believe that doing so may bring misfortune [11-13].

Differences in Asian and non-Asian views about death and dying could potentially influence health preference measurements in several ways. First, in general, Asians may view death and other health-related decisions as family rather than personal matters, in contrast to Caucasians who may value individualism and autonomy [14]. As such, Asians are more likely to value health by taking their families' needs into consideration. Second, Asians, notably the Japanese, generally prefer not to be a burden to others [15]. Hence, they are more likely than Caucasians to assign higher values for the health state of being dead and lower values to those health states in which they are dependent on others (e.g. confined to bed) [16]. By highlighting these cultural differences, we are not implying that views on death are clearly demarcated between Asians and Caucasians. Rather, these important cultural differences suggest that health preferences generated from Caucasian populations may not fully reflect health preferences among Asians and therefore may not be suitable for use in healthcare decision making in Asia. An understanding of the Asian perception towards death is also necessary for handling and interpreting logically inconsistent values in health preferences [17], because the logical order of health states (from worst to best) may be different in different cultures. An understanding of terms used to describe the worst possible health state is also germane in this context, in particular as the term "pits", which has been used in health preference studies, is a British colloquial term which may not be well-understood in this Asian population.

The aims of this study were thus to characterize ease in discussing death (EID) and its influence on health preference measurement and to determine the acceptability of various descriptors of death and "pits"/"all-worst" in health valuation in a multi-ethnic Asian population. We characterised EID and its influence on health preference measurement by evaluating subjects' EID and explored the influence of sociocultural variables on EID. We also studied the influence of EID on VAS/TTO utilities with and without adjustments for sociocultural variables, as this could impact on health preferences and might therefore need to be adjusted for in health valuation studies. We determined the acceptability of various descriptors of death and "pits"/"all-worst" in health valuation and explored the influence of sociocultural variables on the acceptability of these descriptors (of death, pits and all-worst). We studied the appropriateness of these commonly used descriptors because they represent alternative lower anchors for the continuum of health in health valuation studies, with perfect health representing the upper anchor. Hence, the choice of words to describe these health states could potentially influence health preference measurements.

## Methods

### Subjects

In this Institutional Review Board approved study, in-depth interviews in either English or the subject's mother-tongue (i.e. Chinese, Malay or Tamil) by interviewers of the same ethnic group were conducted among consenting Chinese, Malay and Indian Singaporeans (distribution in the general population: 78% Chinese, 14% Malay, 7% Indians; % English-speaking only: Chinese – 16%, Malays – 2%, Indians: 22%; % Bilinguals: Chinese – 32%, Malays: 20%, Indians: 55%) with at least 6 years of education. The various mother-tongue versions of the questionnaire were translated based on the English version. To achieve adequate representation, 2 male subjects (one speaking English, the other his respective mother tongue) and 2 female subjects (one speaking English, the other her respective mother tongue) from each age band (20–29, 30–39, 40–49, 50–59, >60) were recruited from the general population, giving a minimum of 20 subjects per ethnic group.

### Study design

This study was conducted in 3 stages. First, subjects were asked to comment on and rate, using a 0 to 10 horizontal VAS, (1) EID (VAS anchors: most comfortable vs. least comfortable) and (2) self-reported religiosity (a potential determinant of EID; measured in response to the question, "On a scale of 0 to 10, how religious do you feel yourself to be?").

Second, subjects were asked about their views regarding death, several descriptors of death and "pits"/"all-worst". To facilitate the discussion, interviewers prompted subjects with questions such as "How comfortable are you with discussing death?", "Do you think it is a taboo to discuss death?", "Do you believe in life after death?". Subjects' comments were recorded verbatim. Subjects were also asked to comment on and rate, using a 0 to 10 horizontal VAS, (1) acceptability of eight commonly used descriptors of death, i.e. "dead", "passed away", "death", "deceased", "demised", "departed", "sudden death" and "immediate death" (VAS anchors: most acceptable vs. least acceptable) and (2) appropriateness of "pits" and "all-worst" (VAS anchors: most appropriate vs. least appropriate) in describing the worst possible health state (the descriptors were shown on two separate cards).

Third, subjects completed a simple health valuation exercise to determine their preferences for 3 hypothetical EQ-5D defined health states using a 0 to 10 vertical VAS (anchors: best imaginable health state vs. worst imaginable health state) followed by the TTO method. Each health state on the EQ-5D consists of one of 3 possible levels from each of 5 single-item health dimensions. Perfect health on the EQ-5D would be described as 11111 while the worst possible EQ-5D health state would be described as 33333. The 3 health states used in this study were selected from those used in the EQ-5D MVH protocol [18] representing mild (11122), moderate (23321) and severe (32313) impairments. Sociodemographic information was collected using a standardised form. Self-report of chronic medical conditions was determined using a list including diabetes mellitus, hypertension, heart diseases, stroke, asthma or other lung diseases, cancer, rheumatism, back pain or other bone or muscle illness, and mental illness (including depression, anxiety neurosis, schizophrenia).

### Data and statistical analyses

Subjects' characteristics were compared using Fisher's exact or Chi-squared tests for categorical variables or Kruskal-Wallis test for continuous variables, where appropriate. Key points from verbatim records of subjects' comments on (1) EID, (2) acceptability of descriptors of death and (3) appropriateness of "pits" and "all-worst" were summarised.

To study the influence of sociocultural variables including sociodemographic and clinical variables and self-reported religiosity on (1) EID, (2) acceptability of descriptors of death and (3) appropriateness of "pits" and "all-worst", we assessed the relationships between these variables in univariable analyses using Mann-Whitney or Kruskal-Wallis tests (categorical independent variables) or Spearman's correlation (continuous independent variables). Independent variables with p < 0.10 in univariable analyses were then entered into the multiple linear regression (MLR) models. Due to the small number of subjects, we considered the results of MLR analysis exploratory.

To determine the influence of EID on VAS/TTO utilities without adjustment for sociocultural variables for each health state, we assessed the relationships between these variables using Spearman's rank correlation. To determine the influence of EID on VAS/TTO utilities with adjustment for sociocultural variables, we planned MLR in two steps. First, separate preliminary MLR models with EID and a single sociocultural variable as independent variables were created for each health state. Hence, for each health state, a total of eight models were generated, one for each sociocultural variable investigated. Second, a final MLR model with EID and multiple sociocultural variables were created for each health state. Only those sociocultural variables with p < 0.10 from the preliminary models were included in this final model. Data were analysed with STATA [19].

## Results

### Response rate and subject characteristics

Of 69 subjects approached, 63 (91%) participated, two declined participation because they were busy and four declined after hearing that the survey was a discussion on death. None of the subjects terminated the survey prematurely, although they had been informed that they had the freedom to do so. Distribution of subject characteristics and responses are given in Table 1. By design, there was approximately equal number of subjects from each ethnic group, and from both genders. As compared to Malay and Indian subjects, Chinese subjects reported more years of education (p = 0.019). Overall religiosity was moderate (median religiosity scores (IQR): 6.0 (5.0, 8.0)). As compared to Malay and Indian subjects, Chinese subjects reported lower religiosity (median religiosity scores (IQR): 5.0 (2.3, 7.0) vs. 6.5 (5.0, 8.8) vs. 7.0 (5.0, 10), p = 0.036).

**Table 1:** Subject Characteristics and Distribution of Responses by Ethnicity

	**Median (interquartile range), unless otherwise specified**				
	**All (n = 63)**	**Chinese (n = 22)**	**Malays (n = 20)**	**Indians (n = 21)**	p value
Age (years)	44 (32, 56)	45 (32, 56)	42 (26, 50)	35 (41, 57)	0.76
					
Female (N, %)	33 (52)	12 (55)	10 (50)	11 (52)	0.96
					
Years of education	10.0 (8.0, 13.0)	13.0 (10.0, 15.0)	10.0 (8.0, 12.0)	10.0 (8.0, 12.0)	0.019
					
Presence of chronic medical conditions^† ^(N, %)	32 (51)	10 (45)	9 (45)	13 (62)	0.46
					
Working (N, %)	38 (60)	10 (45)	13 (65)	15 (71)	0.19
					
Healthcare background (N, %)^‡^	9 (15)	6 (27)	2 (11)	1 (5)	0.11
					
Religiosity^§^	6.0 (5.0, 8.0)	5.0 (2.3, 7.0)	6.5 (5.0, 8.8)	7.0 (5.0, 10)	0.036
					
Ease in discussing death^§^	8.0 (6.0,10)	8.0 (7.0, 9.0)	8.0 (5.0, 10)	8.0 (6.0, 10)	0.82
					
Acceptability of descriptors of death^§^					
Passed away	8.0 (7.0, 10)	8.0 (7.0, 9.0)	9.5 (7.0, 10)	8.0 (6.5, 10)	0.37
Departed	8.0 (5.0, 9.0)	8.0 (7.0, 9.0)	8.0 (5.0, 10)	6.0 (4.0, 9.5)	0.31
Deceased	8.0 (5.0, 10)	7.0 (5.8, 8.3)	9.0 (7.0, 10)	7.0 (5.0, 10)	0.20
Demised	7.0 (5.0, 10)	7.5 (7.0, 9.0)	8.0 (5.0, 10)	6.0 (3.5, 9.5)	0.27
Death	7.0 (5.0, 9.0)	6.5 (5.0, 8.3)	7.5 (5.0, 10)	5.0 (4.0, 8.5)	0.34
Dead	7.0 (5.0, 9.0)	6.5 (5.0, 8.3)	9.5 (5.0, 10)	6.0 (4.0, 8.0)	0.084
Sudden death	5.0 (3.0, 7.0)	6.0 (3.8, 7.0)	7.0 (5.0, 10)	4.0 (1.5, 6.5)	0.035
Immediate death	5.0 (2.0, 8.0)	5.0 (3.0, 7.3)	6.5 (3.5, 10)	3.0 (0.5, 7.0)	0.033
					
Appropriateness of descriptors of worst health state^§^					
Pits	4.0 (0, 8.0)	5.0 (0, 7.3)	2.5 (0, 5.0)	6.0 (1.0, 8.5)	0.17
All-worst	7.0 (5.0, 9.0)	7.5 (4.8, 8.0)	5.0 (1.3, 7.0)	9.0 (7.0, 10)	<0.001
					
Preferred descriptor of worst health state (N, %)^||^					0.43
Pits	12 (19)	5 (23)	5 (25)	2 (10)	
All-worst	40 (63)	14 (64)	12 (60)	14 (67)	
Neither	11 (16)	3 (12)	3 (15)	5 (24)	
					
Health state ratings^#^					
Visual analogue scale (range 0 to 10)					
Mild (11122)	8.0 (7.0, 10)	7.8 (6.4, 8.0)	9.0 (8.0, 10)	9.0 (7.5, 10)	0.005
Moderate (23321)	4.0 (2.0, 5.0)	3.5 (2.4, 5.0)	0 (0, 0.5)	3.0 (1.5, 5.0)	0.19
Severe (32313)	1.0 (0, 2.5)	1.5 (1.0, 2.5)	0 (1.0, 3.0)	0 (0, 5.0)	0.003
Time-trade off scores (range – 19 to 1)					
Mild (11122)	0.85 (0.45, 0.95)	0.88 (0.56, 0.95)	0.90 (0.50, 1)	0.80 (0.20, 0.95)	0.43
Moderate (23321)	0.00 (-0.67, 0.50)	0.20 (-0.49, 0.75)	-0.10 (-3.00, 0.50)	-0.11 (-0.55, 0.00)	0.07
Severe (32313)	-0.25 (-0.82, 0.05)	0.00 (-0.67, 0.36)	-0.25 (-4.00, 0.06)	-0.33 (-0.75, 0.00)	0.24

IQR – interquartile range. ^†^Self-reported chronic medical conditions included diabetes mellitus, hypertension, heart disease, asthma or lung diseases, bone or muscle illnesses and mental illnesses. ^‡^Details of subjects working in healthcare-related industry – Chinese: 5 pharmacy students, 1 teaching assistant in pharmacy department of a university; Malays: 1 office assistant in pharmacy department of a university, 1 cleaner in healthcare institution; Indian: 1 hospital inpatient care assistant. ^§^Self-reported on a 0 to 10 Likert-type scale, where a higher score indicates higher religiosity/greater ease in discussing death/higher acceptability/greater appropriateness. ^||^p value calculated only for subjects who stated a preference. Among all subjects, all-worst was significantly preferred over pits (p < 0.001). # Each EQ-5D health state consists of 5 dimensions (mobility, self-care, usual activities, pain/discomfort, anxiety/depression) and 3 levels (1 – no problem, 2 – moderate problem, 3 – severe problem). Health state 11122: No problems in walking about; no problems with selfcare; no problems with performing usual activities; moderate pain or discomfort and moderately anxious or depressed. Health state 23321: Some problems in walking about; unable to wash or dress oneself; unable to perform usual activities; moderate pain or discomfort; not anxious or depressed. Health state 32313: Confined to bed; some problems with self-care; unable to perform usual activities; no pain or discomfort; extremely anxious or depressed.

List of original items used in the study questionnaire:

1. Ease in Discussing Death: This piece of medical information mentions about the risk of dying from a new treatment. Are you comfortable with the idea of talking about death? Why? Prompting questions: Why do you find it difficult to talk about death? Do you fear death? For example, do you avoid thinking about death? Do you believe in life after death?

2. Descriptors of Death: In some health surveys, we need to discuss about death. Some words associated with death that may be found in health surveys include 'death', 'dead', 'passed away', 'deceased', 'departed', etc. In your opinion, on a scale of 0 to 10, how acceptable is each of these words shown on this card? Why?

3. Descriptors of Worst Health State: We have a card here that describes a certain health state. Some people have called it the "pits" state. Some people have called it the "all-worst" state. What does the word 'pits' mean to you? Why? What does the word 'all-worst' mean to you? Why? On a scale of 0 to 10, how suitable is the word 'pits' for describing this health state? On a scale of 0 to 10, how suitable is the word 'all-worst' for describing this health state? Which word do you prefer for describing this health state? Can you think of better suggestions for describing this health state? You may want to make reference to the list of terms that I have here on this card.

### Ease in discussing death

Subjects were generally comfortable with discussing death (median EID (IQR): 8.0 (6.0 to 10.0)), with no ethnic differences noted (p = 0.82, Table 1). As education was the only sociocultural variable that significantly influenced EID in univariable analyses (Spearman's correlation coefficient = 0.25, p = 0.045, Table 2), MLR was not performed.

**Table 2:** Univariable Analyses of the Influence of Sociocultural Variables on Ease in Discussing Death

	Ease in discussing death^† ^(Median, IQR/Spearman's Correlation Coefficient)	p value
Ethnicity		0.82
Chinese	8.0 (7.0, 9.0)	
Malays	8.0 (5.0, 10)	
Indians	8.0 (6.0, 10)	
		
Age (years)	-0.094	0.47
		
Gender		0.57
Male	7.5 (7.0, 10)	
Female	8.0 (6.0, 10)	
		
Education (years)	0.25	0.045
		
Chronic medical conditions		0.32
No	8.0 (7.0, 10)	
Yes	8.0 (5.3, 10)	
		
Working		0.69
No	8.0 (7.0, 9.5)	
Yes	7.5 (5.8, 10)	
		
Healthcare background		0.55
No	8.0 (7.0, 10)	
Yes	8.0 (5.5, 9.0)	
		
Religiosity^†^	0.042	0.74

IQR – interquartile range. ^†^Self-reported on a 0 to 10 Likert-type scale, where a higher score indicates greater ease in discussing death/higher religiosity.

### Ethnic differences in health state ratings

Compared to Malays and Indians, Chinese subjects assigned significantly lower VAS scores for the mild health state (median VAS scores: 9.0 vs. 9.0 vs. 7.8, p = 0.005) but significantly higher VAS scores for the severe health state (median VAS scores: 0 vs. 0 vs. 1.5, p = 0.003). There were no statistically significant ethnic differences in TTO scores assigned to any of the three health states.

### Influence of ease in discussing death on health utilities without and with adjustment for sociocultural variables

Correlations between EID and health utilities for the 3 assessed health states were generally weak for all subjects (range: VAS: -0.23 to 0.07; TTO: -0.14 to 0.11, Table 3) and among individual ethnic groups, with the exception of Malay subjects in whom EID showed a moderate correlation with the moderately impaired health state measured using VAS (but not TTO).

**Table 3:** Correlation between Ease in Discussing Death and Health Utilities

	Spearman's Rank Correlation Coefficients Ease in discussing death^†^			
	All	Chinese	Malays	Indians
Health state^‡^				
Visual analogue scale (range 0 to 10)				
Mild (11122)	0.061	0.39	-0.17	0.15
Moderate (23321)	0.070	0.16	0.46	-0.26
Severe (32313)	-0.23	-0.081	-0.27	-0.23
Time-trade off (range -19 to 1)				
Mild (11122)	0.11	-0.038	0.17	0.16
Moderate (23321)	-0.040	-0.28	0.22	0.029
Severe (32313)	-0.14	-0.19	0.13	-0.27

^† ^Self-reported on a 0 to 10 Likert-type scale, where a higher score indicates greater ease in discussing death.

^‡ ^Each EQ-5D health state consists of 5 dimensions (mobility, self-care, usual activities, pain/discomfort, anxiety/depression), each with 3 levels (1 – no problem, 2 – moderate problem, 3 – severe problem).

In the preliminary MLR models including a single sociocultural variable, ethnicity was the only sociocultural variable with p < 0.10 for the moderately impaired health state measured using VAS (Table 4). Hence, the final MLR model was not generated.

**Table 4:** Analyses of the Influence of Ease in Discussing Death (EID) and a Single Sociocultural Variable on VAS Scores in Separate Multiple Linear Regression Models for Moderately Impaired Health State (23321)

Model	**VAS Score (Regression coefficient, 95% confidence interval)**	**p value**
(a) EID and Ethnicity		
EID	-1.85 (-3.92, 0.21)	0.077
Ethnicity^†^		
Malays	-0.15 (-10.75, 10.45)	0.98
Indians	-10.78 (-21.21, -0.35)	0.043
		
(b) EID and age		
EID	-1.75 (-3.85, 0.35)	0.10
Age per year	0.07 (-0.22, 0.36)	0.63
		
(c) EID and gender		
EID	-1.94 (-4.07, 0.19)	0.073
Female^†^	2.06 (-6.87, 10.98)	0.65
		
(d) EID and education		
EID	-1.98 (-4.21, 0.24)	0.080
Education per year	0.18 (-1.53, 1.88)	0.84
		
(e) EID and chronic medical conditions		
EID	-1.84 (-3.99, 0.31)	0.092
Presence of chronic medical conditions^†^	2.02 (-7.00, 11.04)	0.66
		
(f) EID and work status		
EID	-1.90 (-4.03, 0.23)	0.079
Working^†^	1.30 (-7.83, 10.42)	0.78
		
(g) EID and healthcare background		
EID	-2.11 (-4.27, 0.05)	0.055
With healthcare background^†^	0.79 (-11.96, 13.54)	0.90
		
(h) EID and religiosity		
EID	-1.94 (-4.02, 0.15)	0.068
Religiosity^‡ ^(per point on 0–10 VAS)	-1.25 (-2.83, 0.33)	0.12

^†^Reference categories in multiple linear regression were Chinese, male, absence of chronic medical conditions, not working and without healthcare background.

^‡^Self-reported on a 0 to 10 Likert-type scale, where a higher score indicates higher religiosity.

### Discussion on death and acceptability of descriptors of death

Subjects' responses to standardised questions regarding death were as follows:

#### (A) Comfort level in describing death

Over half of our subjects (32/63) felt comfortable with discussing death, verbalizing that death is "natural", "it happens to everyone" or "once you are born, you have to die", etc. One 81-year old Chinese female said that discussing death was not problematic because she was already very old. Another 30-year old Chinese male was comfortable discussing death because "death seemed to be quite far away from me". Five (8%) subjects specified that they were not comfortable discussing death. Among them, one 56-year old Chinese female said that she would be uncomfortable discussing death if this were not a survey. One 62-year old Malay male felt that death cannot be discussed, and another 45-year old Malay male said that he was uncomfortable discussing death because he wanted to live longer.

#### (B) Taboo to discuss death

Only one Chinese (5%) and two Malay subjects (10%) felt it was a taboo to discuss death.

#### (C) Fear of death

All except 5 (25%) Malay subjects (3 males and 2 females) said they did not fear death. These five subjects did not explain why they feared death.

#### (D) Belief in life after death

Interestingly, many subjects (31/63) said they believed in life after death. Two Indian and three Malay subjects said that their religious beliefs influenced their views on death. For four of these subjects, self-reported religiosity was high (range 7 to 10). The fifth subject gave a religiosity score of 5.0.

Although most subjects were comfortable with discussing death, they felt some descriptors of death were more acceptable than others (Table 1). In general, "passed away", "departed" and "deceased" were the most well-accepted descriptors while "sudden death" and "immediate death" were the least well-accepted. Ethnic differences in acceptability of "sudden death" (p = 0.035) and "immediate death" (p = 0.033) were observed, with Indian subjects finding these descriptors less acceptable than Chinese or Malay subjects.

In univariable analyses, one or more sociocultural variables influenced acceptability of six descriptors of death (p < 0.10, Table 5) except "passed away" and "death". However, in multivariable analyses only the presence of chronic medical condition remained significantly associated with acceptability of "departed" (regression coefficient (95% confidence interval, CI): -1.3 (-2.6, -0.053), p = 0.042).

**Table 5:** Univariable Analyses of the Influence of Sociocultural Variables on Descriptors of Death.

	Acceptability of ^† ^(Median, IQR/Spearman's Correlation Coefficient)							
	
	Dead	p value	Passed Away	p value	Death	p value	Deceased	p value
Ethnicity		0.084		0.37		0.34		0.20
Chinese	6.5 (5.0, 8.3)		8.0 (7.0, 9.0)		6.5 (5.0, 8.3)		7.0 (5.8, 8.3)	
Malays	9.5 (5.0, 10)		9.5 (7.0, 10)		7.5 (5.0, 10)		9.0 (7.0, 10)	
Indians	6.0 (4.0, 8.0)		8.0 (6.5, 10)		5.0 (4.0, 8.5)		7.0 (5.0, 10)	
								
Age (years)	-0.017	0.90	-0.021	0.87	-0.11	0.37	-0.11	0.38
								
Gender		0.23		0.40		0.21		0.24
Male	7.0 (5.0, 10)		8.5 (6.8, 10)		7.0 (5.0, 10)		8.0 (5.0, 10)	
Female	6.0 (5.0, 9.0)		8.0 (7.0, 10)		6.0 (5.0, 8.0)		7.0 (5.5, 9.5)	
								
Education (years)	0.030	0.81	0.080	0.53	0.19	0.13	0.22	0.084
								
Chronic medical conditions		0.69		0.69		0.14		0.12
No	7.0 (5.0, 10)		8.0 (7.0, 10)		7.0 (5.0, 9.0)		8.0 (7.0, 10)	
Yes	6.5 (5.0, 9.0)		8.0 (7.0, 10)		5.0 (5.0, 9.0)		7.0 (5.0, 10)	
								
Working		0.65		0.62		0.45		0.52
No	7.0 (5.0, 9.0)		8.0 (7.0, 10)		7.0 (5.0, 9.5)		8.0 (6.5, 10)	
Yes	6.0 (5.0, 10)		8.0 (6.8, 10)		6.5 (5.0, 8.3)		7.0 (5.0, 10)	
								
Healthcare background		0.75		0.44		0.73		0.26
No	6.0 (5.0, 10)		8.0 (7.0, 10)		7.0 (5.0, 9.0)		7.0 (5.0, 10)	
Yes	7.0 (4.0, 9.0)		8.0 (6.0, 9.5)		7.0 (4.0, 8.5)		8.0 (7.0, 10)	
								
Religiosity^†^	0.034	0.79	0.052	0.69	-0.058	0.65	0.088	0.49

	Acceptability of ^† ^(Median, IQR/Spearman's Correlation Coefficient)							
	
	Demised	p value	Departed	p value	Sudden Death	p value	Immediate Death	p value

Ethnicity		0.27		0.31		0.033		0.033
Chinese	7.5 (7.0, 9.0)		8.0 (7.0, 9.0)		6.0 (3.8, 7.0)		5.0 (3.0, 7.3)	
Malays	8.0 (5.0, 10)		8.0 (5.0, 10)		7.0 (5.0, 10)		6.5 (3.5, 10)	
Indians	6.0 (3.5, 9.5)		6.0 (4.0, 9.5)		4.0 (1.5, 6.5)		3.0 (0.5, 7.0)	
								
Age (years)	-0.07	0.58	-0.15	0.25	-0.25	0.05	-0.17	0.2
								
Gender		0.60		0.65		0.32		0.34
Male	8.0 (5.0, 10)		8.0 (5.0, 9.3)		6.0 (4.0, 8.0)		5.0 (3.0, 8.5)	
Female	7.0 (5.0, 9.0)		7.0 (5.0, 9.5)		5.0 (2.5, 7.0)		5.0 (1.0, 7.5)	
								
Education (years)	0.30	0.017	0.25	0.047	0.28	0.024	0.13	0.32
								
Chronic medical conditions		0.53		0.010		0.060		0.080
No	8.0 (6.0, 10)		8.0 (7.0, 10)		6.0 (4.0, 9.0)		5.0 (3.0, 8.0)	
Yes	7.0 (5.0, 9.8)		6.5 (5.0, 8.0)		5.0 (2.0, 7.0)		5.0 (1.0, 7.8)	
								
Working		0.44		0.14		0.56		0.81
No	8.0 (5.5, 10)		8.0 (7.0, 10)		6.0 (4.0, 8.0)		5.0 (2.5, 8.0)	
Yes	7.0 (5.0, 9.3)		5.0 (7.5, 8.3)		5.0 (3.0, 7.0)		5.0 (2.0, 8.0)	
								
Healthcare background		0.27		0.17		0.02		0.09
No	7.0 (5.0, 9.5)		5.0 (5.0, 9.0)		5.0 (3.0, 7.0)		5.0 (2.0, 8.0)	
Yes	8.0 (7.0, 9.5)		8.0 (7.0, 10)		7.0 (6.5, 9.5)		7.0 (5.0, 8.5)	
								
Religiosity^†^	-0.0063	0.96	0.070	0.59	-0.023	0.86	-0.10	0.42

IQR – interquartile range.

^†^Self-reported on a 0 to 10 Likert-type scale, where a higher score indicates greater acceptability/higher religiosity.

### Pits versus all-worst

The majority of subjects (n = 42, 64%) felt that "all-worst" was a better description than "pits" for the worst possible health state on all EQ-5D dimensions (p < 0.001, Table 1). This preference was also reflected in the higher appropriateness scores for "all-worst" versus "pits" (median appropriateness scores (IQR): 7.0 (5.0, 9.0) vs. 4.0 (0, 8.0), p < 0.001). Among subjects who preferred "all-worst", six said they did not know the meaning of "pits". One Malay male said that "pits" sounded like pig and would be offensive. Five Malay subjects (all completing the interviews in Malay) thought "pits" meant graveyard. This suggested that the translation was problematic. However, bilingual subjects in this study were of the opinion that there was no better Malay translation for "pits". Although Malay subjects preferred "all-worst" to "pits", they did not think "all-worst" was very appropriate and suggested using "most terrible" (8/20) instead. Other suggested descriptions for the worst possible health states by all subjects included "most undesirable" (18/63), "most terrible" (13/63) or "worst" (6/63).

In univariable analyses of the influence of sociocultural variables on rating of "all-worst", we found that appropriateness of "all-worst" was rated significantly (i.e. p < 0.10) lower by Malay subjects compared to Chinese or Indian subjects (median appropriateness scores: 5.0 vs. 7.5 vs. 9.0, p < 0.001, Table 6), by those with more years of education (Spearman: -0.23, p = 0.075), or those with healthcare background (median: 6.0 vs. 8.0, p = 0.090). In multivariable analysis of the influence of sociocultural variables on rating of "all-worst", both ethnicity (regression coefficient (95% CI): Malays: -2.7 (-4.5, -0.89), p = 0.004; Indians: 0.83 (-0.92, 2.6), p = 0.35; Chinese as reference) and education (regression coefficient (95% CI): -0.33 (-0.61, -0.049), p = 0.022) remained statistically significant.

**Table 6:** Univariable Analyses of the Influence of Sociocultural Variables on "Pits" and "All-Worst".

	Acceptability of ^† ^(Median, IQR/Spearman's Correlation Coefficient)			
	
	Pits	p value	All-Worst	p value
Ethnicity		0.17		<0.001
Chinese	5.0 (0, 7.3)		7.5 (4.8, 8.0)	
Malays	2.5 (0, 5.0)		5.0 (1.3, 7.0)	
Indians	6.0 (1.0, 8.5)		9.0 (7.0, 10)	
				
Age per 10 years	0.18	0.89	1.00	0.43
				
Gender		0.82		0.83
Male	5.0 (0, 7.3)		7.0 (5.0, 9.3)	
Female	4.0 (0.5, 8.0)		7.0 (4.5, 9.0)	
				
Education (years)	0.025	0.85	-0.23	0.075
				
Chronic medical conditions		0.46		0.22
No	4.0 (1.0, 8.0)		7.0 (5.0, 8.0)	
Yes	3.5 (0, 7.5)		8.0 (5.0, 9.8)	
				
Working		0.09		0.23
No	3.0 (0, 5.0)		7.0 (4.0, 8.0)	
Yes	5.0 (0, 8.0)		8.0 (5.0, 9.3)	
				
Healthcare background		0.46		0.090
No	4.0 (0, 7.5)		8.0 (5.0, 9.0)	
Yes	5.0 (2.5, 8.0)		6.0 (4.0, 7.5)	
				
Religiosity^†^	-0.026	0.84	0.17	0.19

IQR – interquartile range.

^†^Self-reported on a 0 to 10 Likert-type scale, where a higher score indicates greater acceptability/higher religiosity.

## Discussion

In this study among Chinese, Malay and Indian subjects living in Singapore, a multi-ethnic Asian urban state, we characterised ease in discussing death and its influence on health valuation in a multi-ethnic Asian population and determined the acceptability of various descriptors of death and "pits"/"all-worst" in health valuation. We found that subjects were generally comfortable with discussing death. Correlations between EID and VAS/TTO utilities were generally weak, suggesting that EID was unlikely to influence health preference measurement in health valuation studies. We also found that among eight descriptors of death, "passed away", "departed" and "deceased" were the most well-accepted and "sudden death" and "immediate death" were the least well-accepted. The majority of subjects felt that "all-worst" was a better description than "pits" for the worst possible health state.

Our findings are important in several ways. First, to the best of our knowledge, this is the first study to evaluate EID and its influence on health valuation. Our findings suggest that EID is unlikely to affect participation rate (since very few subjects declined participation and none terminated the study prematurely) and cross-cultural comparability of, or to introduce response biases due to unwillingness to discuss death in health valuation studies in Singapore. They also provide a basis and baseline for comparison with similar studies in other socio-cultural contexts.

Second, our finding that sociocultural variables influenced acceptability of several descriptors of death and subjects' assessment of appropriateness of "all-worst" is important in helping to identify the preferred descriptors for use in health valuation studies. For example, the ideal descriptor of death should be one that is not influenced by any of these sociocultural variables. Descriptors that would satisfy this criterion include "passed away" and "death".

Third, to the best of our knowledge, this is the first study that evaluated cultural differences in EID in a semi-quantitative manner. By asking subjects to rate their EID and acceptability of various descriptors, we were able to identify factors that predict acceptability of these descriptors, thus allowing better designed health valuation studies. Fourth, being the first of such studies in Asia, this study also provides useful empirical data to inform design of future valuation studies in an Asian context.

Several aspects of our findings deserve mention. First, the relatively low acceptability of "immediate death" raises a concern about cross-cultural comparability of health valuation studies using this term, which has been commonly used as a descriptor in previous health valuation studies. Due to its relatively low acceptability in this Asian population, subjects may feel offended and be less willing to participate in or complete such studies. Hence, it might be advisable to replace "immediate death" with other descriptors that were better accepted. Ethnic differences in acceptance of "immediate death" may also introduce a systematic bias. For example, participation rates may be lower, rates of missing data may be higher and preference scores for that health state may be lower among Indian subjects compared to Chinese or Malay subjects. An alternative interpretation of this data is that the low acceptability of "immediate death" suggests that it is an appropriate descriptor for a health state that is to be avoided at all costs. Thus, further studies are required to investigate the impact on measurement of health preferences if an alternative to "immediate death" is used as descriptor in health valuation studies.

Second, we recognize that some descriptors of death may be more suitable in a given situation. For example, sudden death would be an appropriate descriptor in studies involving patients with acute myocardial infarction. However, ethnic differences in acceptability of sudden death may introduce bias, and for this reason it would be more appropriate to use an alternative descriptor, which would not introduce this potential bias, even it is if less medically accurate.

Third, the strong preference for "all-worst" over "pits" provides empirical evidence for using this descriptor in future health valuation studies to be performed in this population. Furthermore, as there is no appropriate translation for "pits" in the Malay language, the use of "pits" should ideally be avoided in such studies. We found interesting data suggesting important ethnic differences in the acceptability of descriptors of death and "all-worst". The reasons for this are not clear, and could be related to cultural differences in perception of the worst possible health state. This could be studied in greater detail in future studies. Nevertheless, it was fairly clear that "pits", a British colloquial term, was poorly understood in this study population.

We recognize several limitations of this study. First, the findings may not be readily generalised to the Singaporean general population. For example, subjects with fewer than 6 years of education were not included in this study. Given that EID is associated with years of education, further studies are needed to know if subjects with fewer than 6 years of education are comfortable with discussing death. Nevertheless, it is unclear if subjects with low literacy can participate in health-state valuation studies. Previous studies found that successful (i.e. non-missing, logical) responses tend to come from younger and/or better educated subjects [20,21].

Second, with regards to acceptability of descriptors of death, the discussion was carried out in a somewhat artificial setting. We did not evaluate the acceptability of these descriptors in the context of actual health valuation studies. As one subject pointed out, she was comfortable with discussing death only because this was a survey. Further studies are needed to evaluate if these descriptors of death remain acceptable in the context of actual health valuation studies. Third, given the sample size of our study, the MLR analyses were exploratory.

## Conclusion

In conclusion, we found in this study that Singaporeans were generally comfortable with discussing death and had clear preferences for several descriptors of death and for "all-worst". EID is unlikely to influence health preference measurement in health valuation studies, which suggests that such studies could be performed in Singapore without concerns about the potential impact of EID on participation rate, accuracy of responses and cross-cultural comparability.

## Abbreviations

EID Ease in discussing death;

MLR multiple linear regression;

SG standard gamble;

TTO time trade-off;

VAS visual analogue scale

## Competing interests

The author(s) declare that they have no competing interests.

## Authors' contributions

JT conceived the study, and participated and provided oversight for its design and coordination. HL Wee, YB Cheung and DM participated in the design and coordination of the study and performed the statistical analysis. SC Li, F Xie, XH Zhang, N Luo and KY Fong participated in the design of the study and its coordination. All authors read and approved the final manuscript.
